# Locating the Association

**Published:** 1885-12

**Authors:** J. W. Russey

**Affiliations:** Rising Fawn, Ga.


### Correspondence.

*Editors Atlanta Medical and Surgical Journal:*

In the October number of the JOURNAL is an article from the pen of Dr. R. J. Nunn on locating the State Medical Association and a proposed plan of drawing the individual members of the profession into the Association.

The fact of the relatively small number of practitioners enrolled as members of the State Society is due to other causes than that to be inferred from the article, viz., the few cities or towns in which it has held its sessions.

Georgia, under the Acts of 1837 *et seq.*, is probably more free from the swarm of quacks and charlatans than any sister Southern State, and yet the proportion of members of the State Society is relatively smaller than in any one of them.

This is not to be construed as an argument against legal enactments controlling medical practice, though I am persuaded that in other States some organization was necessary to bind the members of the regular profession more firmly and also to correct abuses.

Just here allow me to express the opinion that the law of 1837 was a wise one, with slight amendment, and the enactment of 1881 was far from being an improvement.

Now as to the matter in hand. What shall be done?

In the first place locate, if you will, but at some central point most accessible to all parts of the State, or have biennial meetings at some fixed point, and let every alternate one be at some place chosen at the preceding meeting.

In the second place encourage the organization of county or district societies, and make the State Society essentially a representative body, basing representation upon membership of county or district society.

By these two steps combined it is possible to assemble annually two hundred or three hundred of the most able representative practitioners in the State.

This estimate is based upon a representation of one for every five members of county or district society. Delegates thus assembled with the permanent members would be far more desirable than the present manner of meeting with but a small proportion of the active profession.

After the above, would come into play the method proposed by Dr. Nunn, its provisions much more feasible than as originally suggested. The secretary would not have such onerous duties to perform, sending notices directly to secretaries of county organizations, and the purposes answered without further pains.

The finances of the State Society would be materially increased by the payment of dues from the subordinate societies.

The State of Georgia having nearly two thousand physicians, and yet the State Association is confined to less than three hundred practitioners, should cause a blush to suffuse the cheek of every member of the profession.

One reason of this state of things is that we country practitioners have come to regard the State Association as a creature of cities. Make membership in State Association dependent upon previous membership in county societies, and this feeling will be overcome, and the result will be that all will feel interested and benefited, the State Association flourish, and instead of a membership of 15 per cent. of the regular profession, it would shortly reach 50 per cent. or more.

J. W. RUSSEY, M. D.

Rising Fawn, Ga., October 14, 1885.

